# Manifestations and outcomes of nocardia infections: Comparison of immunocompromised and nonimmunocompromised adult patients

**DOI:** 10.1097/MD.0000000000012436

**Published:** 2018-10-05

**Authors:** Julie Steinbrink, Joan Leavens, Carol A. Kauffman, Marisa H. Miceli

**Affiliations:** a Department of Internal Medicine; b Department of Infectious Diseases, University of Michigan Healthcare System; c Department of Infectious Diseases, VA Ann Arbor Healthcare System, Ann Arbor, MI.

**Keywords:** immunocompromised patients, nocardia, nocardia infections, nocardiosis, nonimmunocompromised patients

## Abstract

*Nocardia* is a ubiquitous environmental pathogen that causes infection primarily following inhalation into the lungs. It is generally thought to cause infection primarily in immunocompromised patients, but nonimmunocompromised individuals are also at risk of infection. We sought to compare risk factors, clinical manifestations, diagnostic approach, treatment, and mortality in immunocompromised and nonimmunocompromised adults with nocardiosis.

We studied all adults with culture-proven *Nocardia* infection at a tertiary care hospital from 1994 to 2015 and compared immunocompromised with nonimmunocompromised patients. The immunocompromised group included patients who had a solid organ transplant, hematopoietic cell transplant (HCT), hematological or solid tumor malignancy treated with chemotherapy in the preceding 90 days, inherited immunodeficiency, autoimmune/inflammatory disorders treated with immunosuppressive agents, or high-dose corticosteroid therapy for at least 3 weeks before the diagnosis of nocardiosis.

There were 112 patients, mean age 55 ± 17 years; 54 (48%) were women. Sixty-seven (60%) were immunocompromised, and 45 (40%) were nonimmunocompromised. The lung was the site of infection in 54 (81%) immunocompromised and 25 (55%) nonimmunocompromised patients. Pulmonary nocardiosis in immunocompromised patients was associated with high-dose corticosteroids, *P* = .002 and allogeneic HCT, *P* = .01, and in nonimmunocompromised patients with cigarette smoking, bronchiectasis, and other chronic lung diseases, *P* = .002.

Cavitation occurred only in the immunocompromised group, *P* < .001. Disseminated infection was more common in the immunocompromised, *P* = .01, and was highest in solid organ transplant recipients, *P* = .007. Eye infection was more common in nonimmunocompromised patients, *P* = .009. Clinical signs and symptoms did not differ significantly between the 2 groups. The initial treatment for most patients in both groups was trimethoprim-sulfamethoxazole with or without a carbapenem. All-cause 1-year mortality was 19%; 18 (27%) immunocompromised and 3 (7%) nonimmunocompromised patients died, *P* = .01.

Immunocompromised patients with nocardiosis had more severe disease and significantly higher mortality than nonimmunocompromised patients, but clinical presentations did not differ.

## 1 Introduction

*Nocardia* species, filamentous, branching, aerobic gram-positive, weakly acid-fast bacilli, are ubiquitous in the environment. Infection primarily manifests as pulmonary involvement because the main portal of entry is by inhalation.^[1–6]^ However, *Nocardia* species also cause primary cutaneous infection by direct inoculation^[7]^ and can disseminate to many organs, with particular predilection for the central nervous system (CNS).^[8,9]^

*Nocardia* species are usually thought to be opportunists, causing infections mostly in immunocompromised patients.^[10–20]^ In several large series from tertiary care centers, approximately 80% of patients were immunocompromised.^[2–4,20]^ However, a few reports have noted that immunocompetent hosts comprised between 40% and 60% of cases^[5,6]^; most of these patients have underlying conditions, such as chronic lung disease, diabetes, and alcoholism.

In reporting our experience over the last 21 years, we sought to understand differences in risk factors, clinical presentation, diagnostic approach, treatment, and outcomes among patients who are immunocompromised and those who are not.

## 2 Methods

### 2.1 Patients and setting

This retrospective study was conducted at the University of Michigan Health System, a 1000-bed tertiary care hospital in southeastern Michigan. Adult patients 18 years of age and older who were cared for from April 1994 to July 2015 were eligible for inclusion in the study if they were documented to have had at least one culture from blood, body fluid, or tissue that yielded a *Nocardia* species, had compatible symptoms and signs of infection, and their medical record was available for review. One case was included that had been previously reported.^[21]^ This study was approved by the University of Michigan Institutional Review Board (IRBMED).

Patients were stratified into immunocompromised and nonimmunocompromised groups. The immunocompromised group included patients who had HIV infection with CD4 count <200 cells/μL, solid organ or hematopoietic cell transplant (HCT), hematological or solid tumor malignancy treated with chemotherapy in the preceding 90 days, collagen vascular disease or a systemic inflammatory disease treated with immunosuppressive agents in the preceding 90 days, or high-dose corticosteroid therapy (equivalent of ≥0.3 mg/kg/day of prednisone) given for at least 3 weeks before the diagnosis of *Nocardia* infection.

### 2.2 Study design

Data collected from the electronic medical record included patient demographics, comorbid illnesses, type of immunosuppression, clinical features, concomitant opportunistic infections, laboratory and radiological studies, treatment, and outcomes. Concomitant infections were defined as any bacterial, viral, or fungal infection identified at the same time or within 14 days of the diagnosis of *Nocardia* infection. Disseminated *Nocardia* infection was defined as having more than 1 noncontiguous organ involved and/or at least 1 blood culture that yielded a *Nocardia* species.

### 2.3 Microbiology

Nocardia were identified to the species level by phenotypic methods using biochemical assimilation and utilization until early 2014. After that, matrix-assisted laser desorption/ionization – time-of-flight (MALDI-TOF) mass spectrometry was used for identification. For 2 isolates, the species was determined by gene sequence analysis using 16 s ribosomal RNA. Susceptibility testing was performed at Mayo Medical Laboratories, Rochester, MN.

### 2.4 Statistical analysis

We conducted descriptive data analyses for all variables. *t* test, Fisher exact test, and 1-way analysis of variance (ANOVA) were used to determine differences between groups. All statistical analyses were completed using SPSS software, version 24.0 (SPSS, Inc., Chicago, IL).

## 3 Results

### 3.1 Demographics and underlying illnesses

We identified 121 patients who had blood, body fluid, or tissue that yielded a *Nocardia* species in culture from April 1994 to July 2015; of these 121 patients, 112 met the criteria for inclusion and medical records were available for review. The mean age was 55 ± 17 years, and 54 (48%) were women (Table 1). Among these 112 patients, 67 were immunocompromised (60%) and 45 (40%) were not immunocompromised. The most common underlying immunocompromising condition was high-dose corticosteroids, present in 41 (61%) of the immune compromised group. There were 19 solid organ transplant recipients who had received a kidney (9), lung (8), or heart (2) transplant and 16 HCT recipients, 15 of whom had received an allogeneic transplant (Table 1). No patients with HIV infection were found in the study cohort.

**Table 1:**
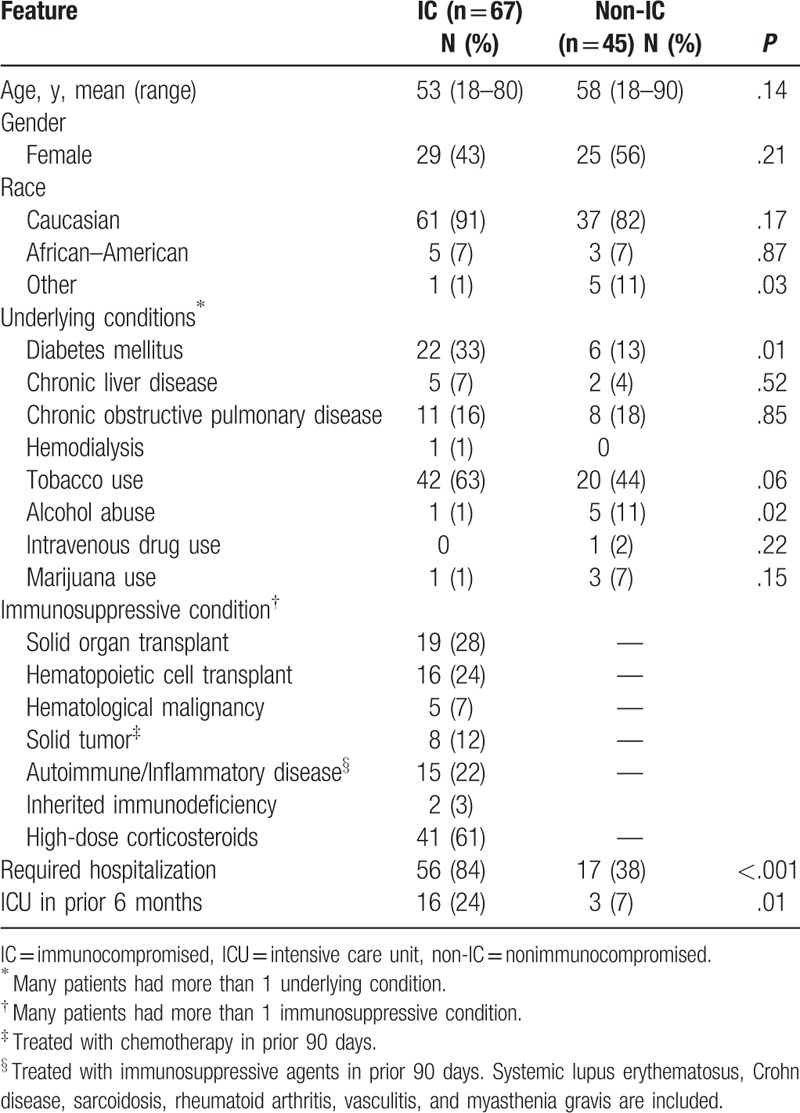
Demographics and underlying conditions in 112 patients with nocardiosis.

Many underlying conditions were similar in the patients who were immunosuppressed and those who were not (Table 1). However, significantly more immunocompromised than nonimmunocompromised patients were hospitalized, *P* < .001 and had been admitted to an intensive care unit in the preceding 6 months, *P* = .01, reflecting more severe illness in this group.

### 3.2 Sites of infection

The lung was the most common site of infection in both immunocompromised and nonimmunocompromised patients (Table 2). In the immunocompromised group with pulmonary involvement, the prominent risk factors were high-dose corticosteroids, *P* = .002 and allogeneic HCT, *P* = .01. Among nonimmunocompromised patients, cigarette smoking and a history of bronchiectasis or other chronic lung disease in 31 of the 45 patients (69%) was significantly associated with pulmonary nocardiosis, *P* = .002.

**Table 2:**
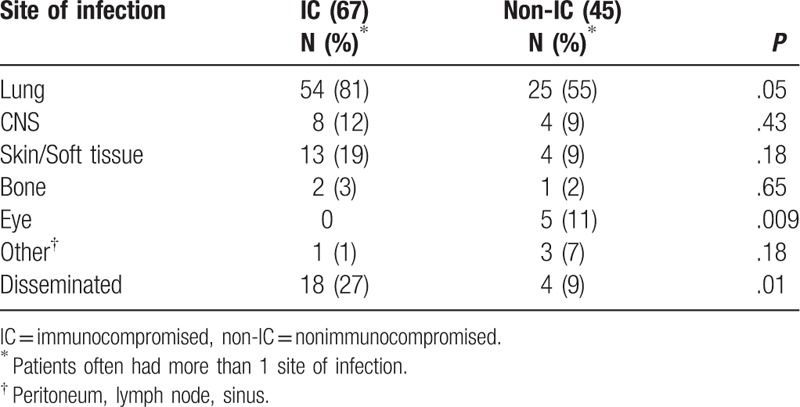
Site of infection in 112 patients with nocardiosis.

As might be expected, disseminated infection was more common in the immunocompromised group, *P* = .01. The highest risk group for dissemination was solid organ transplant recipients, *P* = .007. The primary focus was thought to be lung in almost all cases, and dissemination was most often to the CNS. In only 3 patients, dissemination was documented to skin or subcutaneous tissues, and in another 2 patients, dissemination involved multiple lymph node groups. Only rarely were other viscera or osteoarticular structures involved. Only 4 nonimmunocompromised patients, 2 of whom had central line associated bloodstream infections, had disseminated *Nocardia* infections.

Eight of the 12 (67%) patients who had documented CNS infection were immunocompromised. Ten of the 12 patients had concomitant pulmonary nocardiosis. One nonimmunocompromised patient who presented solely with CNS infection had an episode of “aspiration pneumonia” treated several months before the diagnosis of CNS nocardiosis and, thus, possibly also had prior pulmonary nocardiosis. A single patient had an infected ventriculo-peritoneal shunt.

Of the 17 patients who had skin and/or subcutaneous tissue nocardiosis, 13 (76%) were immunocompromised. Eleven of these 13 infections were characterized by nodules or abscesses that required debridement or drainage, including 4 patients who had experienced a prior fall that damaged the tissue in which the infection later developed and 3 patients in whom the abscess developed following a surgical procedure. Only 3 patients had primary inoculation nocardiosis due to documented penetrating trauma, and 2 others likely had localized inoculation nocardiosis, but a discrete traumatic event was not defined.

Eye involvement occurred only in nonimmunocompromised patients who had experienced trauma to the eye. All 5 patients had corneal ulcerations, 3 from contact lens abrasions and 2 from a traumatically introduced foreign body.

### 3.3 Clinical manifestations

Symptoms and signs depended on the site of infection and did not differ significantly between those who were immunocompromised and those who were not (Table 3). Fever at presentation to the hospital was documented in only 37% of patients. The majority of patients who had pulmonary nocardiosis had dyspnea, cough, and sputum production; hemoptysis and pleuritic chest pain were uncommon. Septic shock occurred in 5 patients, all of whom were immunocompromised; 4 of these patients had disseminated infection and 1 had severe pneumonia.

**Table 3:**
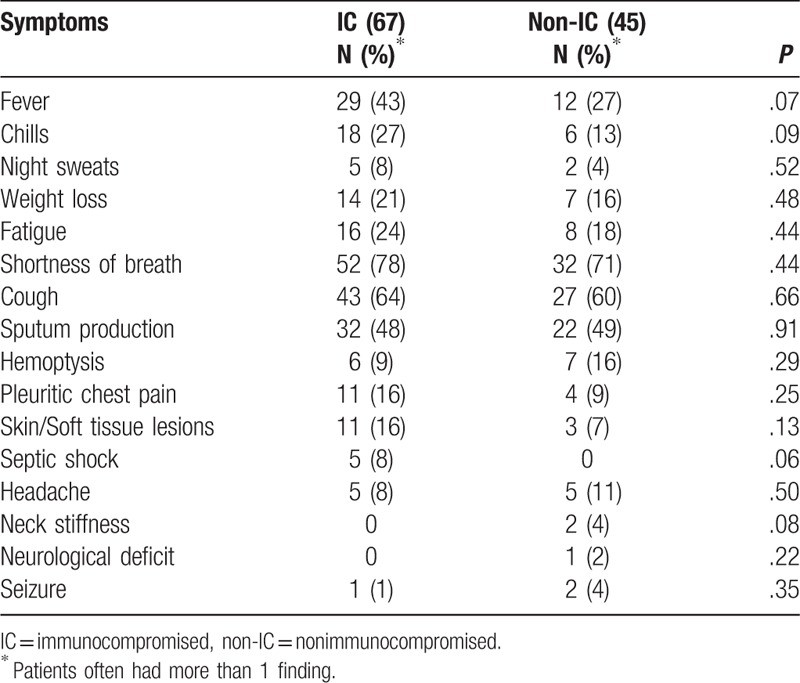
Clinical manifestations in 112 patients with nocardiosis.

Of the 12 patients who had documented CNS nocardiosis, 3 denied any CNS symptoms, 5 had mental status changes, and 4 had headache. Only 1 patient had a focal neurological deficit and 3 had seizures.

Skin lesions, noted in only 3 immunocompromised patients and 2 nonimmunocompromised patients, were described as papules, ulcerations, or cellulitis. Only 1 patient was noted to have a sporotrichoid pattern develop on her arm.

### 3.4 Diagnostic studies

#### 3.4.1 Microbiology

The diagnosis of nocardiosis was established by culture in all patients in this study. The highest yield (71%) was from bronchoalveolar lavage (BAL) fluid and/or sputum; skin lesions and subcutaneous fluid collections yielded *Nocardia* species in 16 (14%) of patients. Blood cultures yielded *Nocardia* species in 7 immunocompromised and 3 nonimmunocompromised patients. In 2 patients in each group, *Nocardia* species were isolated only from blood. One immunocompromised patient clearly had pulmonary infiltrates on chest radiograph, but cultures were not obtained from sputum and bronchoscopy was not performed before she died. The other 3 patients had central line associated bloodstream infections due to *Nocardia* species. Other sites that yielded *Nocardia* species in 1 or 2 patients each were lymph nodes, peritoneal dialysis fluid, sinus aspirate, and bone.

*Nocardia* were identified to species level in all but 5 cases. *Nocardia asteroides* was identified as the organism causing 67% of infections in the immunocompromised group and 73% in the nonimmunocompromised group (Table 4). *Nocardia farcinica* was next most common, and all other species were identified in only 1 or 2 patients in each group. In blood cultures, the species identified were *N. asteroides* in 7 patients and *N. farcinica* in 3 patients. *Nocardia brasiliensis* was associated with skin and soft tissue infections in 3 of the 4 patients in whom it was isolated. All species except *Nocardia caviae*, *Nocardia nova*, and *Nocardia cyriacigeorgia,* which only caused pulmonary infection, were able to cause disseminated infection.

**Table 4:**
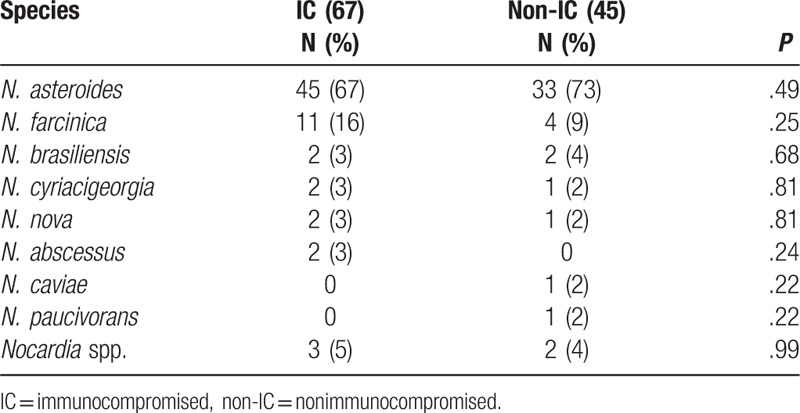
*Nocardia* species isolated from 112 patients with nocardiosis.

Gram stain was performed in 90 samples and revealed Gram-positive, beaded, filamentous organisms typical of *Nocardia* species in 42 (47%). Modified acid-fast stain was performed less often (72 samples) and was positive in only 7 samples (10%).

Antimicrobial susceptibilities were performed on 54 isolates. No isolates were resistant to linezolid, and only 1 isolate (*N. asteroides*) was resistant to trimethoprim-sulfamethoxazole (TMP-SMX). Three isolates were resistant to imipenem, and 2 of these also were resistant to amikacin. Resistance was higher for all other tested antibiotics: tetracyclines (13%), ceftriaxone (28%), moxifloxacin (30%), and clarithromycin (56%).

#### 3.4.2 Other laboratory studies

The white blood cell (WBC) count was >10,000 cells/μL with >60% neutrophils in 34 of 57 (60%) immunocompromised patients and 18 of 31 (58%) nonimmunocompromised patients. No patient had neutropenia (<500 neutrophils/μL). Absolute lymphocyte counts <500 cells/μL were noted in 19 of 50 (38%) immunocompromised patients and 10 of 28 (35%) nonimmunocompromised patients.

#### 3.4.3 Radiology

There were few differences noted in CT imaging of the thorax between the 2 groups with the exception of cavitation and bronchiectasis (Table 5). Cavitation occurred only in the immunocompromised group, *P* < .001, and most often in those who had received an allogeneic HCT and had graft-versus-host disease. Ten of the 21 (48%) nonimmunocompromised patients had bronchiectasis compared with 5 of the 51(10%) immunocompromised patients, *P* < .001. Nodules were noted in most patients, irrespective of immune status; fewer had ground glass opacities and consolidation.

**Table 5:**
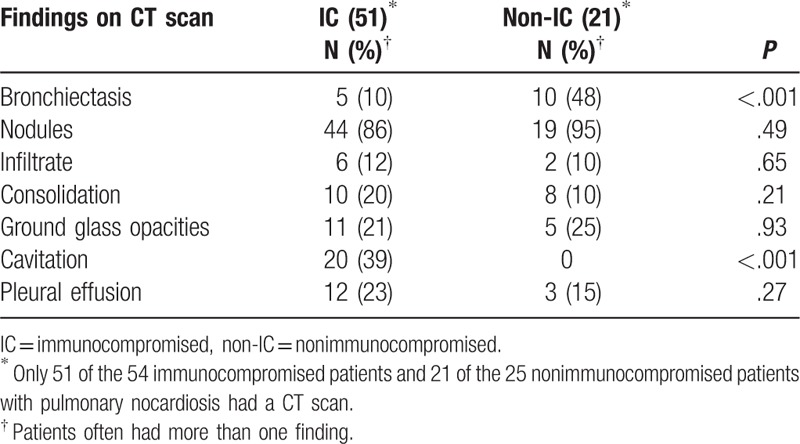
Radiographic findings in 72 patients with *Nocar*dia pulmonary infection.

All 12 patients who had CNS nocardiosis had a CT and/or MRI study performed; in 3 patients who had no CNS symptoms, the diagnosis of nocardial brain abscess was made by an MRI study. Four patients had multiple abscesses and 3 patients had a single abscess documented on CT or MRI. Four other patients had less well-defined multiple enhancing lesions on MRI, and the patient with an infected shunt had no abnormalities on CT scan.

### 3.5 Concomitant infections

Concomitant infections occurring within 14 days of the diagnosis of nocardiosis were found in 38 patients (57%) who were immunocompromised and 21 (47%) who were not (Table 6). Pulmonary aspergillosis was the most common concomitant infection in both groups and occurred in patients who had pulmonary nocardiosis. Similarly, infection with nontuberculous mycobacteria occurred in patients who also had pulmonary nocardiosis. Even though bronchiectasis was significantly more frequent in the nonimmunocompromised population, nontuberculous mycobacterial infections were seen in both groups. With the exception of 1 patient, viral infections were found only in immunocompromised patients; cytomegalovirus viremia or disease was noted in 8 (21%) of the 38 immunocompromised individuals who had concomitant infections, 5 of whom were allogeneic HCT recipients.

**Table 6:**
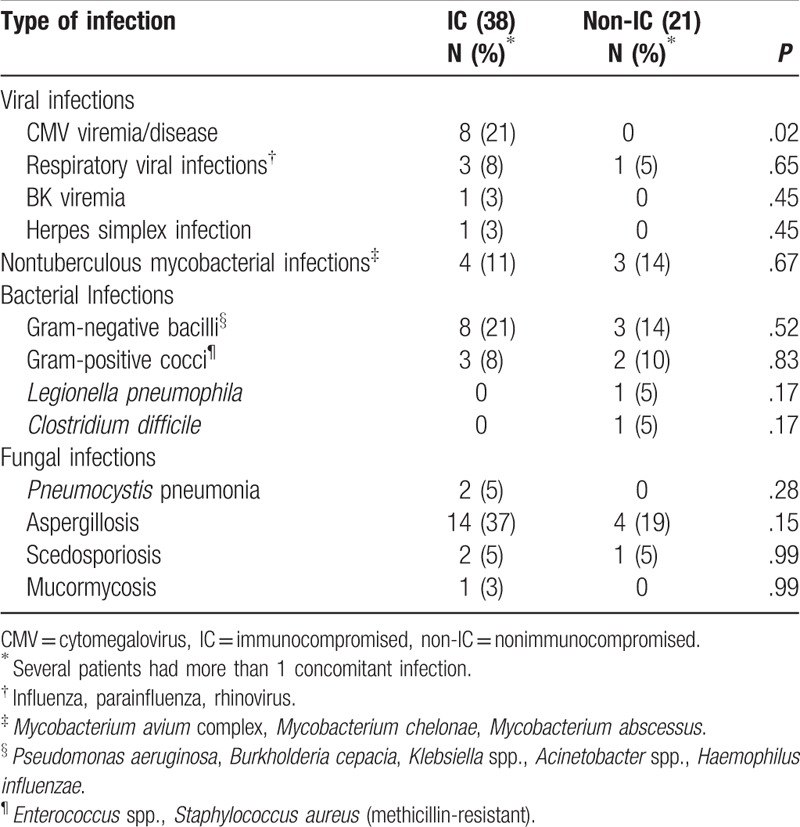
Concomitant infections that occurred within 14 days of the diagnosis of nocardiosis in 59 patients.

### 3.6 Treatment

Infection was initially attributed to another entity and not to nocardiosis in 56 (50%) patients, irrespective of whether they were immunocompromised or nonimmunocompromised. Most patients were treated initially for typical bacterial causes of community-acquired or hospital-acquired pneumonia. One patient was empirically treated for tuberculosis, and 3 patients were empirically treated for fungal pneumonia.

The initial treatment regimen for nocardiosis for most patients in both immunocompromised and nonimmunocompromised groups consisted of trimethoprim-sulfamethoxazole (TMP-SMX) with or without a carbapenem. Forty-seven of the 67 (70%) immunocompromised patients received TMP-SMX; in 26 patients (55%), this was an oral formulation. Sixteen of the 45 (36%) nonimmunocompromised patients received TMP-SMX; for 14 (87%) patients, this was given orally. In 11 (16%) immunocompromised patients, TMP-SMX was combined with either imipenem or meropenem, and in another 3, with amikacin. Combination therapy was given to only 2 nonimmunocompromised patients.

Imipenem or meropenem was used alone or combined with amikacin in 12 immunocompromised patients and 2 nonimmunocompromised patients. Other agents, including ceftriaxone, minocycline, and linezolid, were used for initial therapy in only 7 patients in the immunocompromised group and 5 patients in the nonimmunocompromised group.

For 8 immunocompromised patients, treatment was not given because a decision was made to initiate hospice care, the diagnosis was not made before the patient died, or they were transferred to another hospital without the culture result being acknowledged by their physician and follow-up was lost. Excluding the 5 patients who had eye infections, there were 10 nonimmunocompromised patients for whom treatment could not be verified; most were lost to follow-up, and their physician did not comment upon the culture results.

Of the 44 immunocompromised patients and 19 nonimmunocompromised patients who had long-term follow-up at our medical center, the duration of therapy with oral TMP-SMX, minocycline, or a fluoroquinolone was similar. Treatment continued for 269 ± 171 days among immunocompromised patients and 284 ± 178 days among nonimmunocompromised patients.

Ten immunocompromised patients (15%) were receiving TMP-SMX for *Pneumocystis* or other prophylaxis at the time that they developed infection with *Nocardia.* Eight were lung or heart transplant recipients, 1 had received an allogeneic HCT, and 1 had chronic granulomatous disease. The usual dosage was 1 double-strength tablet 3 times weekly. They all developed *Nocardia* pneumonia, 2 had dissemination to the CNS, and 1 also had a soft tissue abscess. The organisms remained susceptible to TMP-SMX.

### 3.7 Outcomes

All-cause 1-year mortality was 19%; 18 (27%) immunocompromised patients died compared with 3 (7%) nonimmunocompromised patients, *P* = .01. Eight of the 18 immunocompromised patients died of nocardiosis. The site of infection in these 8 patients was lung in 4 and disseminated infection in 4, 3 of whom had a positive blood culture. Five of these patients died less than 2 weeks after diagnosis, and 3 died within 1 to 4 months after diagnosis. Only 1 death, in a patient with CNS infection, was attributed to nocardiosis among patients who were nonimmunocompromised.

## 4 Discussion

The importance of *Nocardia* species as opportunistic pathogens in immunocompromised patients has been more completely studied than in patients who do not have underlying immunosuppression Our review of patients highlighted some important differences between these 2 groups. Immunocompromised patients were significantly more likely to experience dissemination, have documented bloodstream infection, require hospitalization, and die of *Nocardia* infection.

However, we also found that many aspects of infection with *Nocardia* species were similar, irrespective of the state of immune suppression of the host. The clinical manifestations of nocardiosis did not differ significantly between those who were immunocompromised and those who were not, a finding similar to that noted by Kim et al.^[20]^ We noted an almost even distribution of men and women in both groups, in keeping with several recent reports,^[5,6]^ but differing from others who reported the classic distribution of nocardiosis occurring as much as 70% of the time in men.^[2–4,12,20]^

Immunity against infection with *Nocardia* species involves multiple components of the immune system acting in concert with one another.^[9]^ Neutrophils, the first line of defense, partially inhibit growth but are not able to effect clearance of *Nocardia* from tissues.^[22,23]^ T-lymphocytes have both a direct action on *Nocardia* and most importantly activate macrophages to kill *Nocardia*.^[24,25]^ Corticosteroids, present in 61% of our patients who were immunocompromised and 37% of all patients, are known to suppress T-cell function and appeared to be a major risk factor associated with nocardiosis. The proportion of patients who have received corticosteroids has varied from 21% to 62% in other smaller series of nocardiosis in tertiary care hospital populations.^[2–5,20]^

The lung was the major target organ involved in infection with *Nocardia* species in both the immunocompromised and nonimmunocompromised group. This has been true in most reported series^[4–6,9]^ and is not surprising because exposure is generally thought to be via inhalation of the organisms from the environment. Bronchiectasis has been associated with pulmonary nocardiosis, and in some centers appears to be an increasing association, especially in women.^[6]^ Recent series focusing on patients who had only pulmonary nocardiosis without dissemination to other organs found chronic pulmonary disease, chronic liver disease, and diabetes as major risk factors.^[26,27]^ In 1 series, 1 species, *Nocardia cyriacigeorgica,* was prominently associated with pulmonary infection and poor outcomes.^[28]^ In our patients, as well as others previously reported, cavitation was significantly associated with immunosuppression.^[20]^

Whether dissemination ensues following respiratory tract infection is dependent on containment of the initial infection by the host. With obvious immunosuppression, such as with high-dose corticosteroids, solid organ transplantation, or HCT, we found dissemination to be common, as have others.^[3,6,12,17,20]^ However, patients who were presumed healthy also were found to have disseminated infection, especially to the CNS, as noted in other reports.^[8,9]^ It is possible that a yet unknown or rare underlying defect in host response is present in such patients. The recent report of the presence of anti-GM-CSF antibody as a risk factor for nocardiosis may explain some cases of disseminated infection, including 1 patient who had dissemination in our study.^[21,28]^

CNS infection, present in 12 patients, was silent in 3 patients and found only by MRI study. The absence of specific CNS symptoms and signs is uncommon, but has been noted by others.^[8,9]^ For this reason, it seems reasonable to image the CNS in any patient who has been found to have forms of nocardiosis other than localized inoculation infection.

Localized cutaneous infection acquired by inoculation was uncommon in our cohort. This form of nocardiosis, which is often caused by *N. brasiliensis*, has been reported more commonly from tropical and subtropical areas.^[5,7]^ Our patients were more likely to have had subcutaneous and other soft tissue abscesses that were secondary to prior surgical procedures or nonpenetrating trauma several weeks before. In all of these cases, the growth of *Nocardia* species from deep tissues or abscess fluid was not expected.

*N. asteroides,* followed by *N. farcinica*, were the most commonly identified *Nocardia* species based on phenotypic analyses. However, major changes have occurred in the taxonomy of the *Nocardia* genus in the last decade, due to the advances in the use of molecular techniques.^[29,30]^ Currently, 16srRNA gene sequencing is the preferred method of identification, but MALDI-TOF mass spectrometry will likely assume an increasing role in identification of *Nocardia* species in the next decade.^[30,31]^ At last count, there were 54 species of *Nocardia* that can cause disease in humans and that fit into 6 major taxa: *N. abscessus, N. nova complex, N. farcinica, N. brevicatena/N. paucivorans, N. transvalensis complex,* and *N. cyriacigeorgica.*^[30]^ Interestingly, *N. asteroides*, the most common species noted in this and most other clinical reviews, is now rarely identified in the clinical laboratory.^[29]^

Both immunocompromised and nonimmunocompromised patients were treated primarily with TMP-SMX, and in vitro susceptibility testing confirmed that all isolates, with the exception of one *N. asteroides*, were susceptible to this agent. This is in disagreement with a study from the Centers for Disease Control and Prevention (CDC) that reported that 42% of isolates representing multiple species that had been submitted to the CDC from 1995 to 2004 were resistant to TMP-SMX.^[32]^ Studies from other countries and those that studied more recent isolates have not found TMP-SMX resistance to be common and have noted that resistance is species dependent.^[33,34]^ For example, Larruskain et al^[33]^ noted that among isolates from Spain, *N. farcinica* and *N. carnea* had TMP-SMX resistance rates of 42% and 58%, respectively, but most other species were susceptible. Schlaberg et al,^[34]^ testing recent isolates from the United States, found TMP-SMX resistance in only 2% of 1299 isolates, and all but one of these isolates were *N. pseudobrasiliensis* or *N. transvalensis* complex. These variances in susceptibility testing point out that it is essential to identify *Nocardia* organisms to the species level and to do susceptibility testing by approved techniques. Currently, TMP-SMX remains the agent of choice, often combined with a carbapenem in seriously ill patients.

Fifteen percent of immunocompromised patients in our study were on TMP-SMX for prophylaxis at the time that they were diagnosed with nocardiosis. Our results corroborate the findings of others who have noted that TMP-SMX, given for *Pneumocystis* prophylaxis at dosages ranging from 1 single-strength tablet daily or several times weekly to 1 double-strength tablet 2 to 3 times weekly, is not adequate to prevent nocardiosis.^[4,10,12,35,36]^ Whether higher dosages of TMP-SMX could prevent *Nocardia* infection in immunosuppressed patients is not known.

Our study has several limitations. It is a retrospective single-center study, which can limit the ability to generalize the results to other institutions and populations. The taxonomy of the genus *Nocardia* has undergone major revisions in the last decade. We used the species name that was documented in the chart. However, it is likely that, using current molecular techniques, many different species would have been identified and far fewer would have been labeled as *N. asteroides*.

A strong aspect of our study is that we cared for a sufficient number of patients who were nonimmunocompromised, as well patients who were immunocompromised, allowing us to assess risk factors, clinical and diagnostic aspects, treatment, and outcomes that differed or were similar between these groups.

## Author contributions

**Conceptualization:** Marisa H. Miceli.

**Data curation:** Joan Leavens.

**Formal analysis:** Marisa H. Miceli.

**Investigation:** Julie Steinbrink, Joan Leavens.

**Methodology:** Marisa H. Miceli.

**Resources:** Joan Leavens.

**Supervision:** Carol A. Kauffman, Marisa H. Miceli.

**Validation:** Carol A. Kauffman.

**Writing – original draft:** Julie Steinbrink, Joan Leavens.

**Writing – review & editing:** Carol A Kauffman, Marisa H. Miceli.
